# Diabetic and non-diabetic patients in the intensive care unit: morbidity and mortality

**DOI:** 10.1186/1758-5996-7-S1-A103

**Published:** 2015-11-11

**Authors:** Antonio Carlos Barros, Íkaro Soares Santos Breder, Antonio Mário Tassinari, Arnaldo Moura Neto, Elizabeth Pavin João, Maria Candida Ribeiro Parisi

**Affiliations:** 1 Universidade Estadual de Campinas, Campinas, Brazil

## Background

Studies made in intensive care wards have associated stress induced hyperglycemia and mortality. Diabetes is a strong predictor of death in patients with Heart Failure.The relation between diabetes, renal disease and mortality has been related to cardiovascular disease (CVD) and hemodynamic instability during hemodialysis.

## Aims

We conducted a prospective observational study in an intensive care unit to evaluate morbidity and mortality in diabetic patients compared to non-diabetics.

## Materials and methods

Between January and December 2013, 97 patients were studied (diabetics and non-diabetics). We analyzed gender, age, total hospitalization days, number of exams (echocardiography, x-ray, computed tomography, magnetic resonance imaging), number of prescribed antibiotics, invasive procedures (dialysis catheter, nasogastric tube, hemodialysis, surgery intervention) and outcome: death, discharged to the ward or home.

## Results

From the 97 patients, 51 were women. Mean age was 54.44 yrs. and mean length of stay 2.24 days. The major cause of admission in both groups was sepsis (39.2%), followed by cardiovascular disease (22.7%). As for outcome, 36.1% were discharged home, 39.2% transferred for the wards, 21.6% died and 3.1% had other destinations. Comparing diabetics and non-diabetics, there were more invasive procedures (55.3 vs 44.6%, p=0.049) and hemodialysis (66.7 vs 33.3%, p=0.03) in the first group. Regarding mortality, 28.3% of diabetic patients died, compared to 15.7% of non-diabetic patients. In patients with diabetes, the number of antibiotics was an independent mortality predictor (p=0.02, OR 2.058; C.I. 1,29-3,284). In the group as a whole, the mortality predictors were age (p=0.044; OR 1.028, C.I. 1.001-1.057) and number of antibiotic used (p<0.001; OR 1.954, C.I. 1.385-2.757).

## Conclusion

As expected in intensive care, in both groups mortality was associated with age and number of antibiotics used. However, in patients with diabetes care should be taken as patients have a higher mortality ratio and often demand more invasive procedures and hemodialysis. Despite not so old, several of these patients may have previous DM related renal impairment.

